# Effects of Pre-exhaustion Versus Traditional Resistance Training on Training Volume, Maximal Strength, and Quadriceps Hypertrophy

**DOI:** 10.3389/fphys.2019.01424

**Published:** 2019-11-19

**Authors:** Thiago Barbosa Trindade, Jonato Prestes, Leônidas Oliveira Neto, Radamés Maciel Vitor Medeiros, Ramires Alsamir Tibana, Nuno Manuel Frade de Sousa, Eduardo Estevan Santana, Breno Guilherme de A. T. Cabral, Whitley Jo Stone, Paulo Moreira Silva Dantas

**Affiliations:** ^1^Graduation Program in Physical Education, Catholic University of Brasília, Brasília, Brazil; ^2^Department of Arts, Federal University of Rio Grande do Norte, Natal, Brazil; ^3^Department of Physical Education, Federal University of Rio Grande do Norte, Natal, Brazil; ^4^Faculdade Estácio de Sá de Vitória, Espírito Santo, Brazil; ^5^Graduation Program in Physical Education, Federal University of Rio Grande do Norte, Natal, Brazil; ^6^School of Nutrition, Kinesiology, and Psychological Sciences, University of Central Missouri, Warrensburg, MO, United States

**Keywords:** training load, training efficiency, pre-fatigue, muscle thickness, resistance exercise, muscle strength

## Abstract

**Background:**

The pre-exhaustion (PreEx) method is used as a resistance training (RT) method to increase muscle mass, yet the chronic effects of this method are poorly understood.

**Objective:**

Although readily prescribed as a RT method for promotion of muscle hypertrophy, few researches give light to gains made after chronic PreEx RT. Therefore, we compared the effects of traditional versus PreEx RT programs on muscle strength, body composition, and muscular hypertrophy in adult males.

**Methods:**

Untrained subjects (age: 31.37 ± 6.83 years; height: 175.29 ± 5.52 cm; body mass: 82.04 ± 13.61 kg; 1RM leg press: 339.86 ± 61.17 kg; 1RM leg extension: 121.71 ± 11.93 kg) were submitted to 9 weeks of RT with weekly sessions. Traditional (TRT) group (*n* = 12) performed three sets at 45° of leg press exercise at 75% of 1RM, PreEx group (*n* = 12) completed a set to failure on a leg extension machine prior to the leg press, and the control (CON) group (*n* = 7) did not train. Maximum strength, muscle thickness, and body composition were analyzed.

**Results:**

PreEx group increased in maximal strength on leg press (16 ± 8%) and leg extension (17 ± 11%), while the TRT group improved by 15 ± 9 and 11 ± 4%, respectively. The thickness of the quadriceps muscles increased for both intervention groups. Specifically, the post-training thickness of the vastus lateralis was significantly higher for PreEx (55%) compared to the CON group. The TRT group presented a greater loss of total and thigh fat mass when compared with the PreEx method. These results were found in the presence of a lower training load for the PreEx group.

**Conclusion:**

The PreEx training can decrease the total training volume while maintaining results in strength and hypertrophy when comparing to TRT. However, TRT may be optimal if the goal is to decrease fat mass.

## Introduction

Resistance training (RT) is an exercise modality recommended for its ability to improve neuromuscular fitness, athletic performance, and increase general health characteristics (Schoenfeld and Grgic, 2017). The manipulation of RT variables, such as the type of exercise, training volume, and rest interval between sets directly affect the magnitude of neuromuscular adaptations (Fonseca et al., 2014; Figueiredo et al., 2017; Fink et al., 2018). The volume–load relationship, for example, defined as the product of repetitions and weight lifted [number of repetitions × load (kg)], is an accepted tool for workload quantification during RT, and has a direct relationship to strength and hypertrophy gains, up to a certain limit (Schoenfeld and Grgic, 2017). Nevertheless, it is possible to observe gains in muscle hypertrophy, and strength with different training workload, considering variables such as the time under tension, and metabolic and mechanical stress, even with lower RT workloads (Schoenfeld et al., 2016).

While multidimensional, it is generally agreed that muscle hypertrophy is positively affected by high levels of muscle tension resulting in hormonal and metabolic response, consequently serving as the stimulus for protein synthesis (i.e., incorporation of new nuclei to muscle fibers, increase the contractile components, and muscle cross-sectional area) (Folland and Williams, 2007; Schoenfeld, 2013). There is a possible association between muscle damage with the increase in protein synthesis; and although not linear, also with muscle hypertrophy (Damas et al., 2016). A part from muscle damage and tension, metabolic stress is an important mediator of hypertrophic adaptations (Schoenfeld, 2013; Goto et al., 2017). It is speculated that once a certain level of mechanical tension is generated, metabolic stress may assume a determinant role in the optimization of hypertrophic responses (Schoenfeld, 2013). To note, hypertrophy is not homogeneous process, proportional along the whole skeletal muscle, or in a group of muscles that share the same proximal/distal insertions (Narici et al., 1989; Wakahara et al., 2013). This effect would be attributed to functional differences of distinct portions of the muscle associated with specific regional activation during exercise (Wakahara et al., 2012; Earp et al., 2015).

Altering training variables potentiate several types of RT methods. The combination of these variables allow for the increase of time under tension that promotes an acute increase in protein synthesis, resulting in cellular signaling to increase strength and muscle size (Tran et al., 2006; Burd et al., 2012; Figueiredo et al., 2017). The continued need to expose athletes to novel stimuli is required for continued results (Prestes et al., 2017; de Almeida et al., 2019). As such, there is justification for evidence to compare alternative RT methods other than traditional strategies (American College of Sports Medicine, 2009; Schoenfeld et al., 2016; Lasevicius et al., 2018). The pre-exhaustion (PreEx) method is a common practice among bodybuilders and RT enthusiasts (Figueiredo et al., 2017). However, the utility and efficacy of PreEx raises contrasting opinions among practitioners and researchers (Carpinelli, 2010, 2013).

Pre-exhaustion is generally implemented through the combination of two or more exercises for the same muscle group, in an almost uninterrupted sequence with the objective to maximize strength and hypertrophy (Brennecke et al., 2009; Fisher et al., 2014). Jones (1971) suggested that the use of compound exercises (those that activate several muscles concurrently) may be limited by the momentary failure of smaller/weaker muscles. This often results in a compensatory strategy of the stronger muscles, underutilizing their potential. Thus, researchers suggest pre-exhausting with exercises that target specific groups (Augustsson et al., 2003; Ribeiro et al., 2018). This specific strategy is not the consensus among all professionals (Gentil et al., 2007; Fisher et al., 2014), and has generated discussions about this RT method (Prestes et al., 2015).

Many projects have attempted to provide evidence on the efficacy of PreEx with acute electromyographic approaches, calculation of total training volume, rating of perceived exertion, and blood lactate measures (Augustsson et al., 2003; Gentil et al., 2007; Brennecke et al., 2009; Guarascio et al., 2016; Soares et al., 2016; Gołaś et al., 2017). These abbreviated studies are not able to determine the adaptation in muscle strength, hypertrophy, or body composition when implemented over time. To date, the authors could find only one study investigating the chronic effects of PreEx. Fisher et al. (2014) found no differences between traditional RT and PreEx in body mass, fat-free mass, and fat mass in a sample of trained men, and women. Aguiar et al. (2015) also utilized knee extension exercise by including a pre-exhaustive set with 20% of one-repetition maximum to failure before high intensity RT, and demonstrated an increase in maximal strength, muscle cross-sectional area, and muscle endurance.

Previous studies did not use exact the PreEx method by failing to implement a standardized exercise selection, rest interval, or load (Carpinelli, 2010). The available evidence about PreEx is limited and inconclusive on its impact on muscle hypertrophy. Thus, the aim of the present study was to compare the effects of traditional resistance training (TRT) versus PreEx programs on muscle strength, body composition, and regional muscle hypertrophy in adult men. We hypothesize that the PreEx method promotes greater magnitudes of muscle hypertrophy and strength compared with the TRT, without differences in body composition.

## Materials and Methods

### Study Design

**FIGURE 1:**
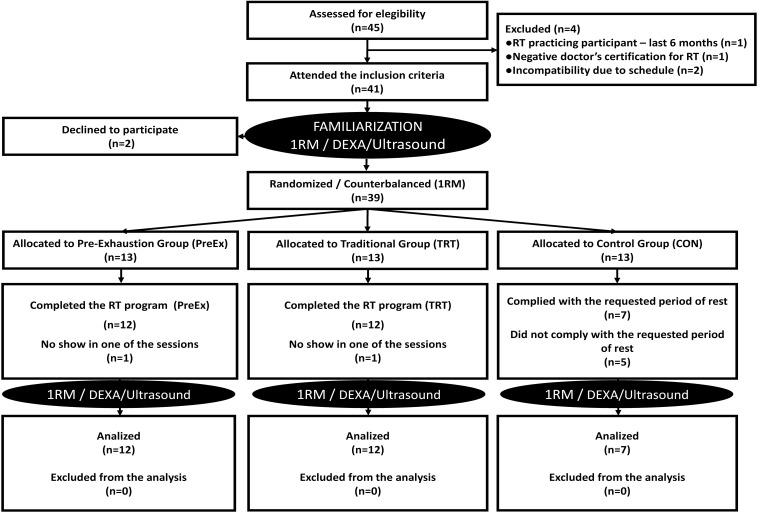
Study flowchart.

This was a prospective, randomized controlled trial designed to compare the effects of TRT versus Pre-Ex methods on maximal strength (1RM), quadriceps and gluteus maximus hypertrophy, body composition (body fat percentage and fat-free mass), and external training load in adult men. Participants completed 1RM tests, muscle ultrasound imaging, and dual-energy X-ray absorptiometry (DXA) before and after 9 weeks of training, which was preceded by nine familiarization sessions, three times per week. The 1RM tests were completed for seated leg extension and 45° leg press. After the first round of tests, participants were randomly distributed in a counterbalanced design (blinded) for leg press 45° 1RM, into three groups: control remained untrained CON (*n* = 7), TRT (*n* = 12), and PreEx (*n* = 12). The tests were repeated within 3 days of completing the experiment (Figure 1).

### Subjects

Apparently healthy men, aged 18–40 years participated after voluntary signing a pre-approved informed consent document. The inclusion criteria were as follows: a minimum 1-year of uninterrupted experience with RT including the RT exercises leg extension and 45° leg press (but not in the 6 months previous to the study), no marks of “yes” on the Physical Activity Readiness Questionnaire, and no contraindications for RT after being examined by a physician. Participants were excluded if presenting with any of the following characteristics: physical disabilities, osteomioarticular limitations, vegetarian diet, use of medication that could affect muscle hypertrophy or training capacity (i.e., anabolic steroids), history of RT in the 6 months leading to the study, presence of diabetes, hypertension, or any other chronic disease. Thirty-one subjects completed the experimental period, obeying the criteria established for the analysis, and the sample size was determined by convenience. The main baseline characteristics of Control (CON), TRT, and PreEx groups are presented in Table 1. All subjects were informed about the procedures, risks, and benefits of the study, and the study was approved by the Ethics Research Committee from the local University (protocol number: 2.110.224).

**TABLE 1:** Participant baseline characteristics.

	**PreEx**	**TRT**	**CON**	***p***
Age (years)	31.3 ± 7.3	31.4 ± 5.5	30.8 ± 10.2	NS
Body mass (kg)	79.8 ± 13.8	81.7 ± 13.8	82.8 ± 17.9	NS
Height (cm)	174.5 ± 4.2	174.4 ± 7.4	176.0 ± 4.4	NS
BMI (kg/m^2^)	26.1 ± 3.7	26.8 ± 3.7	26.6 ± 5.1	NS
1RM leg press (kg)	338.8 ± 60.5	342.1 ± 55.9	335.8 ± 89.9	NS
1RM leg extension (kg)	123.0 ± 9.1	121.4 ± 9.9	117.0 ± 16.1	NS
Relative 1RM leg press	4.3 ± 0.6	4.2 ± 0.8	4.1 ± 0.9	NS

*Results are mean ± SD; PreEx, pre-exhaustion; TRT, traditional; CON, control; BMI, body mass index; NS, non-significant difference between groups; 1RM, one-repetition maximum.*

### Resistance Training Program

During the 9-week intervention or CON, participants from TRT and PreEx trained 2 days per week with three sets to failure (defined as the inability to complete another repetition with appropriate technique), at 75% of 1RM. Exercises were completed on the 45° leg press (Physicus^®^, São Paulo, Brazil) wherein participants were advised to maintain the cadence of 20 repetitions per minute (rpm; 1s concentric: 2s eccentric) with 1 min of rest interval between sets (American College of Sports Medicine, 2009). The PreEx group completed an additional set to failure on the leg extension (Physicus^®^, São Paulo, Brazil), immediately before (≤10 s) the leg press set. The PreEx load for the bilateral leg extension was 20% of 1RM, from 90° to 30° of knee flexion (0° = complete knee extension), with a cadence of 30 rpm (1 s concentric:1 s eccentric), maintained with a metronome. Each training session was prefaced by an exercise specific warm-up on the leg press (1 set of 12 repetitions) using a self-selected load. Training loads were readjusted every 2 weeks based on the most recent 1RM leg press reassessment in the TRT and PreEx groups, and leg extension on the PreEx group. During all training sessions, participants also completed 1 set of 10–12 submaximal repetitions on bench press, dumbbell press, front lat pull-down, seated row, trunk flexion, and extension exercises. The RT sessions were performed between 9:00 p.m. and 12:00 p.m. and each subject was supervised by an experienced professional. Subjects in the CON group were advised to maintain their habitual routines of exercise (soccer, running, jiu-jitsu, and judo were reported). During the intervention, if a subject reported any type of RT, including calisthenics, this person was excluded from control group, which resulted in significant sample loss. Training groups were advised to avoid any other type of exercise.

### Total Training Volume

During all leg press and leg extension sets, subjects were instructed to perform as many repetitions as possible to failure. Thus, the total training volume for TRT and PreEx were not equalized, and were calculated as follows:

– Total training volume − PreEx group: {1 set of leg extension [1 × (load × repetitions)]} + {3 sets of 45° leg press [3 × (load × repetitions)]};
– Total training volume − TRT group: {3 sets 45°of leg press [3 × (load × repetitions)]};
– This procedure was adopted to maintain training methods as they are incorporated during daily training practice, other than equalizing total training volume.

### Nutritional Orientation

Subjects were advised by a nutritionist to complete a dietetic report of 3 days, including a weekend day to analyze their habitual food intake. Each participant was interviewed on three occasions: during the last week of familiarization (before the RT intervention); in the mid-point (during); and after the last RT session (post). The CON group was also interviewed on the same schedule. Standardized portions were used to evaluate the quantity of food and drinks consumed. Total values of energy intake and macronutrients were calculated by using conventional software (Avanutri, version 3.1.4, Rio de Janeiro, RJ, Brazil). In the case of protein ingestion below 1.4 g per kg of body mass, the subject was advised to increase this ingestion to 1.4–2 g per kg of body mass following the guidelines set by the International Society of Sports Nutrition (Jäger et al., 2017). This procedure was adopted to guarantee a positive protein balance and minimize the interference in potential muscle growth. Participants received instruction to maintain their habitual diets during the experimental period (noting the protein recommendation described above) and water was ingested *ad libitum*.

### Familiarization Protocol

Before the first evaluation phase (week A of evaluations), all participants completed a 3-week RT orientation program (three non-consecutive days per week totally nine sessions). This period was designed to familiarize the participants to the RT exercises (regardless of previous experience) to standardize exercise technique, training cadence, and also to minimize the learning effect on the testing protocols (Aguiar et al., 2015). During the first three sessions (days 1, 2, and 3), volunteers completed one set of 12–15 submaximal repetitions of bilateral leg extension and three sets of 12–15 submaximal repetitions on the 45° leg press. In the second week (days 4, 5, and 6), individuals completed 1RM tests for leg press, and in the third week (days 7, 8, and 9), they completed 1RM tests for the leg extension. All familiarization sessions and tests were performed at the same laboratory under controlled temperature, between 9:00 and 11:00 p.m.

### 1RM Testing

Maximum strength was evaluated following standardized 1RM procedures recommended by Brown and Weir (2001). The 1RM tests for the leg extension and leg press (7 days between them in the familiarization weeks) followed the same procedures: general warm-up (5 min of cycle ergometer at light intensity), eight repetitions at 50% of estimated 1RM (according to their performance during the familiarization period). Two minutes later, participants completed three repetitions at 70% of estimated 1RM. After 3 min of rest, they tried their maximum load in one repetition with progressively heavier loads. The 1RM was determined in three attempts, using 3 min of rest, respectively. To assure 1RM reliability before the RT program, the tests were repeated after 72 h recovery. Verbal encouragement was provided during the tests to guarantee the maximum effort. The intraclass correlation was determined between the second and third test day, where the higher load was used as 1RM. The intraclass coefficients were ≥0.99 and ≥0.96 for leg press and leg extension, respectively. Every 3 weeks during the training period tests were repeated to update training loads.

### Muscle Thickness

**FIGURE 2:**
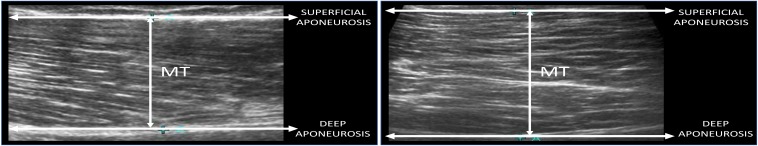
Example of ultrasound image showing the cross-cutting scans of the vastus lateralis muscle – distal part, at 35% of the length of the thigh **(left)** and gluteus maximus **(right)**. MT, muscle thickness.

Before and after the RT programs, muscle thickness from quadriceps femoris (two portions per muscle) were evaluated using ultrasound images gathered from a blinded, experienced physician using ultrasound mode B (Toshiba Aplio Mx, SSA-780 A, Toshiba Medical System^®^), and a probe of 100 mm, set to 10–15 MHz. Resting images were collected at a specific joint angle (150°), corresponding to almost full knee extension (180°). Participants were positioned supine on a stretcher, where they rested for 20 min. After this interval, the transducer was aligned to the fascicular plane to visualize the fascicules on the ultrasound monitor. After the registering of the quadriceps images, the participant moved to prone position to measure gluteus maximus thickness. When the quality of the image was deemed to be satisfactory, the image was saved to the hard drive, and muscle thickness dimensions were determined by measuring the distance between the deep and superficial aponeuroses (Figure 2). All participants were advised to avoid alcoholic drinks and exercise 72 h before the exams. Ultrasound measures were taken 72 h after the last training session to avoid interference. The precision parameters provided by the manufacturer are: image depth scale, track to 280 mm (<±5% or <1 mm, if <20 mm).

The measures were taken distally and proximally to the quadriceps muscles to evaluate possible non-homogeneous alterations in muscle thickness. The measured regions were adopted according to the recommendations from Ema et al. (2013), excluding gluteus maximus.

The measuring sites were determined by the following parameters:

Gluteus maximus (GM, one region): 50% of the sacral vertebra, and the greater trochanter of the femur.
Vastus lateralis (VL, two regions): (distal) 35% of the thigh length from the popliteal crease to the greater trochanter – and (proximal), 55%.
Vastus medialis (VM, two regions): (distal) 15% of the thigh length from the popliteal crease to the greater trochanter – and (proximal), 35%.
Rectus femoris (RF, two regions): (distal) 50% of the thigh length from the popliteal crease to the greater trochanter – and (proximal) 70%.

The measures of muscle thickness were quantified by the software Image J 1.42q (National Institutes of Mental Health, EUA). To test the reliability of the images, 11 subjects repeated the exams 48 h later. The intraclass correlation and coefficient of variation (CV) findings were as follows: GM (0.737 and 5.06%); VL 55% (0.819 and 4.64%); VL 35% (0.918 and 5.93%); VM 35% (0.804 and 8.93%); VM 15% (0.956 and 3.94%); RF 70% (0594 and 5.03%); and RF 50% (0.868 and 4.78%). All images were analyzed by the same operator, a biomedical image specialist (blinded to the participant group assignment).

### Body Composition

Body composition was determined by DXA with a transverse scan from head to feet on the equipment Lunar Prodigy Advance Model (General Electric Company^®^, Boston, MA, United States). Participants were positioned on a stretcher, in dorsal decubitus, where they remained for approximately 5 min to allow for the full body scan. The following variables were analyzed: total body mass (kg); fat-free mass (kg) and percentage fat mass (relative to total body mass); lean mass (kg), and body fat percentage from the lower limb. Body composition from whole body and lower limb was measured by algorithms from the software provided by the DXA device. All participants were advised to maintain normal hydration, avoid alcoholic drinks, and exercise 72 h before the exams. In a methodology review, Nana et al. (2015) observed among studies using DXA for the Assessment of Body Composition in Athletes and Active People, measurements of CV ranging from 0.5 to 2.5% for lean mass and 0.8 to 5.0% for fat mass.

### Statistical Analysis

The data are expressed as mean value and standard deviation (SD). Shapiro–Wilk tests were applied to check for normality distribution of study variables. ANCOVAs were used to determine the effect of the two RT training programs and CON group on post-intervention strength and anthropometric variables after controlling for pre-intervention variables. Paired samples *t*-tests were used to determine the differences between pre- and post-intervention variables for each exercise program. The effect size calculation (ES = difference between pre- and post-intervention divided by pre-intervention SD) and the ES strength training scale proposed by Rhea (2004) were used to evaluate the magnitude of the training effects. The level of significance was *p* ≤ 0.05 and SPSS version 20.0 (Somers, NY, United States) software was used.

## Results

**FIGURE 3:**
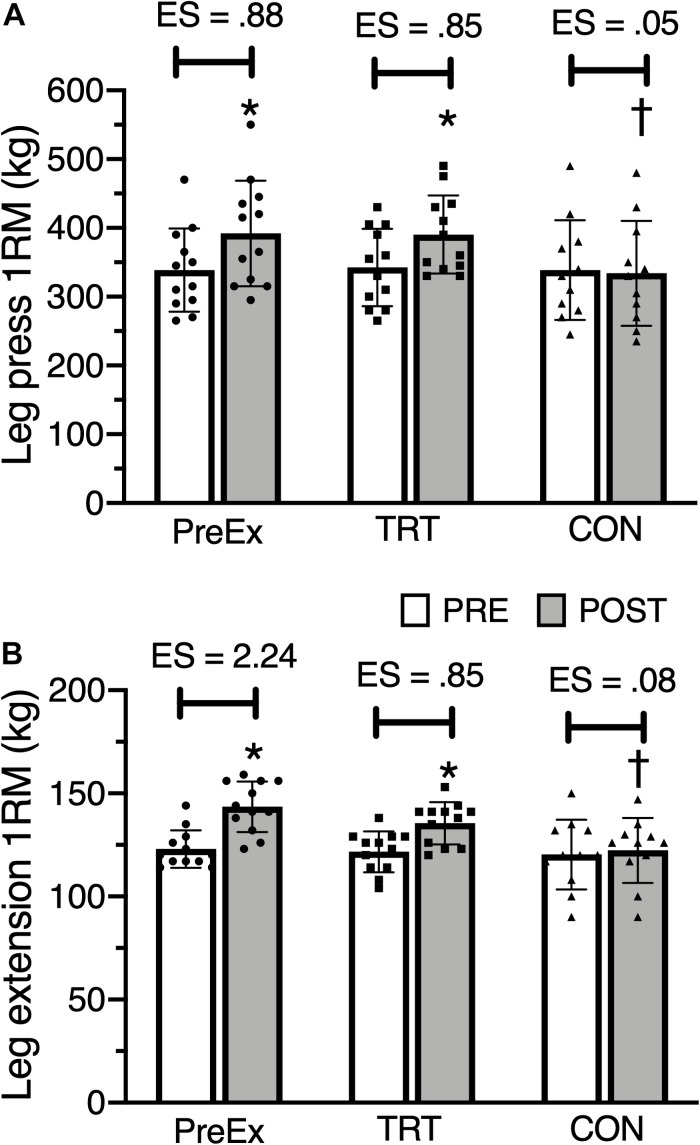
Mean ± SD of one-repetition maximum (1RM) for 45° leg press **(A)**, and leg extension **(B)** pre and post pre-exhaustion (PreEx), traditional resistance training (TRT), and control (CON) groups. ES, effect size; ^∗^*p* ≤ 0.05 for pre-intervention; ^†^*p* ≤ 0.05 for PreEx and TRT after adjustment.

Figure 3 presents 1RM values for 45° leg press and leg extension exercises pre- and post in the PreEx, TRT, and CON groups. After adjustment for pre-intervention 1RM values, there was no statistical difference (*p* > 0.05) in post-intervention variables between groups. The training effect was higher for PreEx group (ES = 2.24-large) than the TRT group (ES = 0.85-small) on leg extension exercise. On 45° leg press exercise, the training effect was similar for PreEx (ES = 0.88-small) and TRT groups (ES = 0.85-small). The 1RM increased in the PreEx group by 16 ± 8% (45° leg press) and 17 ± 11% (leg extension). The TRT group averaged increases of 15 ± 9 and 11 ± 4% for leg press and leg extension, respectively.

Pre and post body composition parameters are shown in Table 2. After adjustment for pre-intervention body composition, there was not a statistically significant difference (*p* > 0.05) in post-intervention between PreEx, TRT, and CON groups, and some ES for PreEx and TRT groups were trivial (Table 2).

**TABLE 2:** Mean ± SD, percentage of change and effect size (ES) of body composition pre and post pre-exhaustion, traditional resistance training, and control groups.

	**Pre**	**Post**	**Change (%)**	**ES**
**Body mass (kg)**				
Pre-exhaustion	79.8 ± 13.8	80.3 ± 14.1	0.6 ± 1.9	0.04 (trivial)
Traditional	81.7 ± 13.8	82.5 ± 14.1	1.0 ± 2.2	0.06 (trivial)
Control	84.8 ± 14.0	85.1 ± 13.2	0.4 ± 1.9	0.02 (trivial)
**Lean mass (kg)**				
Pre-exhaustion	60.5 ± 7.0	61.3 ± 7.1	1.4 ± 2.1	0.12 (trivial)
Traditional	59.0 ± 6.5	60.6 ± 6.6^∗^	2.7 ± 2.6	0.24 (trivial)
Control	61.5 ± 6.8	61.7 ± 6.7	0.4 ± 2.2	0.03(trivial)
**Fat mass (%)**				
Pre-exhaustion	24.3 ± 7.1	23.7 ± 6.8	−2.2 ± 5.5	0.09 (trivial)
Traditional	28.0 ± 7.7	26.8 ± 7.1^∗^	−3.8 ± 4.6	0.16 (trivial)
Control	27.9 ± 5.3	28.0 ± 5.3	0.1 ± 4.3	0.00 (trivial)
**Thigh mass (kg)**				
Pre-exhaustion	25.4 ± 5.1	25.9 ± 5.4	1.7 ± 4.2	0.09 (trivial)
Traditional	26.7 ± 5.2	27.0 ± 4.8	1.4 ± 4.0	0.06 (trivial)
Control	28.1 ± 4.8	27.7 ± 4.4	−1.1 ± 3.8	0.07 (trivial)
**Thigh lean mass (kg)**				
Pre-exhaustion	19.4 ± 3.3	19.9 ± 3.5^∗^	2.5 ± 3.7	0.15 (trivial)
Traditional	19.4 ± 2.4	20.0 ± 2.3^∗^	2.9 ± 3.9	0.22 (trivial)
Control	20.5 ± 2.8	20.3 ± 2.8	−0.7 ± 3.5	0.06 (trivial)
**Thigh fat mass (%)**				
Pre-exhaustion	21.9 ± 5.2	21.5 ± 4.9	−1.7 ± 4.4	0.09 (trivial)
Traditional	25.3 ± 7.0	24.2 ± 6.3^∗^	−3.7 ± 4.4	0.15 (trivial)
Control	25.6 ± 4.5	25.3 ± 4.2	−1.0 ± 4.7	0.06 (trivial)
**BMD (kg)**				
Pre-exhaustion	1.34 ± 0.11	1.35 ± 0.12	0.7 ± 1.8	0.09 (trivial)
Traditional	1.32 ± 0.13	1.30 ± 0.12	−0.9 ± 1.8	0.10 (trivial)
Control	1.30 ± 0.10	1.30 ± 0.10	−0.5 ± 1.6	0.06 (trivial)

*BMD, bone mineral density; ^∗^*p* ≤ 0.05 for pre-intervention.*

After adjustment for pre-intervention, there were no differences in post-intervention hypertrophy (*p* > 0.05) for gluteus maximus, rectus femoris, and vastus medialis thickness between groups (Table 3). However, the post-intervention 55% vastus lateralis thickness was significantly higher (*p* ≤ 0.05) for PreEx (55%) compared with the CON group (55%). Quadriceps muscle thickness increased (*p* ≤ 0.05) for both training groups (excluding gluteus maximus). The training effect, represented by the ES, was large for rectus femoris (50%), vastus lateralis (55%), and vastus lateralis (35%) in the PreEx group, and large change for the rectus femoris (70%) and vastus lateralis (35%) in the TRT group.

**TABLE 3:** Mean ± SD, percentage of change and effect size (ES) of muscle thickness pre and post pre-exhaustion, traditional resistance training, and control groups.

	**Pre**	**Post**	**Change (%)**	**ES**
**Gluteus maximum (mm)**				
Pre-exhaustion	29.1 ± 7.8	33.0 ± 6.2	25 ± 58	0.50 (small)
Traditional	29.1 ± 7.4	34.9 ± 4.8	30 ± 44	0.78 (small)
Control	30.1 ± 4.8	32.5 ± 3.4	10 ± 16	0.52 (small)
**Rectus femoris 70% (proximal) (mm)**				
Pre-exhaustion	21.5 ± 2.7	26.3 ± 3.9^∗^	23 ± 12	1.78 (moderate)
Traditional	20.4 ± 2.4	25.5 ± 3.2^∗^	26 ± 13	2.16 (large)
Control	21.0 ± 3.5	24.3 ± 2.4	17 ± 13	0.95 (small)
**Rectus femoris 50% (distal) (mm)**				
Pre-exhaustion	17.5 ± 3.3	24.9 ± 5.1^∗^	44 ± 26	2.28 (large)
Traditional	18.0 ± 3.0	23.4 ± 3.3^∗^	32 ± 21	1.81 (moderate)
Control	19.1 ± 4.6	20.2 ± 5.8	6 ± 19	0.24 (trivial)
**Vastus lateralis 55% (proximal) (mm)**				
Pre-exhaustion	20.9 ± 3.1	27.4 ± 4.0^∗^	32 ± 18	2.12 (large)
Traditional	21.5 ± 3.4	27.3 ± 4.8^∗^	28 ± 15	1.72 (moderate)
Control	22.5 ± 4.0	24.1 ± 4.2^†^	7 ± 7	0.40 (trivial)
**Vastus lateralis 35% (distal) (mm)**				
Pre-exhaustion	19.5 ± 2.4	27.5 ± 3.0^∗^	43 ± 24	3.34 (large)
Traditional	20.9 ± 3.0	26.9 ± 2.9^∗^	30 ± 16	2.01 (large)
Control	21.4 ± 3.4	22.1 ± 4.6	4 ± 19	0.20 (trivial)
**Vastus medialis 35% (proximal) (mm)**				
Pre-exhaustion	22.8 ± 4.6	28.6 ± 5.7^∗^	28 ± 28	1.27 (moderate)
Traditional	21.4 ± 7.8	26.8 ± 10.0^∗^	25 ± 29	0.69 (small)
Control	26.5 ± 6.3	25.4 ± 5.4	−3 ± 19	0.18 (trivial)
**Vastus medialis 15% (distal) (mm)**				
Pre-exhaustion	23.1 ± 4.5	28.5 ± 5.2^∗^	25 ± 18	1.20 (small)
Traditional	22.4 ± 4.3	28.6 ± 5.4^∗^	30 ± 25	1.42 (moderate)
Control	23.5 ± 5.5	25.3 ± 5.5	8 ± 10	0.31 (trivial)

*^∗^*p* ≤ 0.05 for pre-intervention; ^†^*p* ≤ 0.05 for pre-exhaustion after adjustment.*

**FIGURE 4:**
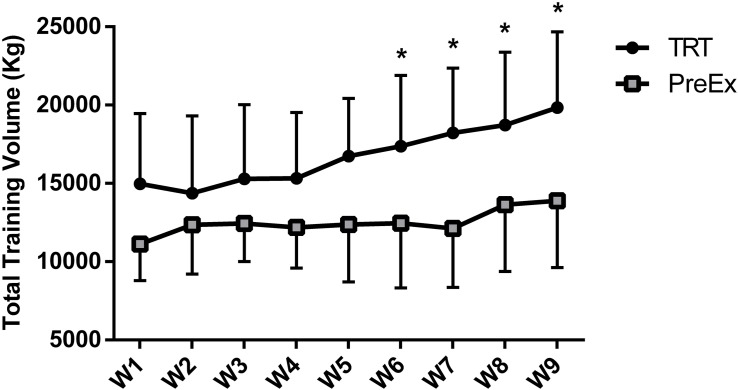
Mean values of total training volume (kg), calculated by load × repetitions × sets. W, week; TRT, traditional resistance training; PreEx, pre-exhaustion. ^∗^Significant difference compared with the PreEx group (*p* < 0.05).

External loads achieved by PreEx and TRT groups during the 9 weeks of intervention are presented in Figure 4. The mean values of total training volume, presented in kilograms (kg), were higher for the TRT group throughout the 9 weeks. The volume was statistically different from the sixth week and beyond: 39.41, 50.37, 37.30, and 42.79%, respectively. The dietetic profiles of the participants did not change during the intervention, and there was no difference between groups for percentage of carbohydrates, proteins, and lipids consumed (Table 4).

**TABLE 4:** Dietetic analysis from PreEx, TRT, and CON groups during the intervention.

	**PreEx**	**TRT**	**CON**	**Group**	**Time**	**Interaction**
	**(*n* = 12)**	**(*n* = 12)**	**(*n* = 7)**			**(G × T)**
**Carbohydrates (g)**						
Pre	275.2 ± 114.2	360.2 ± 211.7	313.6 ± 132.8	0.801	0.274	0.535
During	283.3 ± 109.6	241.5 ± 145.0	308.7 ± 187.9			
Post	253.4 ± 86.2	270.1 ± 91.8	258.7 ± 81.1			
**Protein (g)**						
Pre	121.3 ± 68.1	152.8 ± 47.1	128.5 ± 39.6	0.402	0.330	0.093
During	145.0 ± 65.2	152.2 ± 46.3	144.3 ± 44.9			
Post	160.1 ± 79.6	113.3 ± 27.4	99.2 ± 18.9			
**Lipids (g)**						
Pre	63.0 ± 29.4	91.4 ± 35.3	90.0 ± 30.5	0.323	0.645	0.150
During	90.2 ± 31.9	77.0 ± 25.4	100.6 ± 37.4			
Post	76.4 ± 22.4	88.1 ± 27.4	77.4 ± 18.7			

*PreEx, pre-exhaustion; TRT, traditional resistance training; CON, control. There was no difference between groups.*

## Discussion

The present study compared the effects of PreEx versus TRT on maximum strength, muscle hypertrophy, body composition, and total training volume in adult men. The main findings revealed that, despite the higher total training volume achieved by the TRT group, the gains in muscle strength and hypertrophy were similar between interventions. Both training groups resulted in hypertrophy of different regions of the quadriceps muscles. Only the PreEx group exhibited an increase in vastus lateralis proximal region compared to the CON group. There was no gluteus maximus hypertrophy, regardless of the training method and total training volume. Both groups increased lower limb fat-free mass, while only the TRT group displayed an increase in total body fat-free mass, decrease in total fat body percentage, and lower limb body fat. The TRT and PreEx increased maximum strength on leg press (no difference between them). Maximal strength on leg extension increased more in the PreEx compared with the TRT group. Thus, the initial hypothesis was partially confirmed.

Training volume was not equalized to allow for generalizations to real daily training conditions (Carpinelli, 2010). Although strong evidence supports the idea that higher total training volume directly affects muscle hypertrophy (Figueiredo et al., 2017; Schoenfeld and Grgic, 2017; Grgic et al., 2018), this does not seem to be the case, especially when a RT method is performed under real training set conditions.

Pre-exhaustion training reduced the total training volume despite the additional set of leg extension before 45° leg press. The mean repetitions completed in the leg extension were 56 to failure. Previous literature supports this overall repetition decrease when individuals completed a multijoint exercise immediately after a single joint exercise to fatigue (Augustsson et al., 2003; Gentil et al., 2007). It is speculated that the elevated metabolic stress induced by low load leg extension to failure immediately before the 45° leg press attenuated total training volume possible. This lower training volume with PreEx did not impair the muscles’ hypertrophic response, even when comparing to the higher volume TRT. There is little argument that hypertrophy results from chronic RT and that hypertrophic specific mediators are likely not all identified. However, the potential to grow muscle with low volume RT through PreEx may be a potential exercise prescription for those with little time to train or low training age.

The overall greater training volume prescribed to the TRT group may explain the decrease in body fat and increase in total fat-free mass. It has been reported that caloric deficit is directly proportional to the total workload completed during training sessions (Haff, 2010), and could decrease fat mass. Both training protocols resulted in similar adaptations in lower limb fat-free mass and fat mass, despite the differences noted in total body. These data were supported using the segmented data from DXA and lower limb muscle hypertrophy evaluated by ultrasound imaging. The current investigation differs from available literature, with variance potentially due to their lack of dietetic control, different RT prescription, and the current implementation of more appropriate measurement tools (Fisher et al., 2014).

The results from the muscle thickness analysis confirm the tendency for inhomogeneous hypertrophy of the quadriceps muscle, similar to previous studies (Narici et al., 1989; Akima et al., 2004; Blazevich et al., 2006; McMahon et al., 2014; Earp et al., 2015). Although no differences were observed between training prescription, greater magnitudes of effect were noted for the PreEx group in the distal portion of the rectus femoris, proximal and distal portions of the vastus lateralis, and proximal portion of the vastus medialis. TRT displayed higher magnitudes of thickness for the proximal portion of the rectus femoris, and distal portion of the vastus medialis. A possible explanation for the inhomogeneous alterations in muscle thickness may be attributed to selective recruitment of different regions of the quadriceps due to different exercise intensity (Blazevich et al., 2006) and type of exercise (Earp et al., 2015). It has been speculated that the elevated metabolic stress induced by leg extension with low load (20% 1RM) to failure paired with heavier load 45° leg press could generate hypertrophy in a variety of muscle fibers (type I and type II) (Aguiar et al., 2015; Grgic et al., 2018).

Greater magnitude of change for quadriceps muscle thickness for the PreEx may be attributed to the higher contribution of rectus femoris during leg extension (Ema et al., 2013) and the vastus muscles during multijoint exercises (Escamilla et al., 1998). Fonseca et al. (2014) showed that the inclusion of exercise variation resulted in hypertrophy of all quadriceps heads in untrained subjects, while the exclusive use of the squat with the same training volume was inefficient in rectus femoris hypertrophy.

There appears to be a correlation between specific muscle activation in different regions of a muscle and inhomogeneous hypertrophy (Wakahara et al., 2013). Although speculative, agonists may decrease or maintain their electromyographic activation in multijoint exercises when implementing PreEx (Augustsson et al., 2003; Gentil et al., 2007; Brennecke et al., 2009; Soares et al., 2016). To tease out this hypothesis, future PreEx training studies should consider including the evaluation of metabolic markers, muscle biopsies, intracellular hypertrophy pathways, and more to draw precise conclusions.

No change in gluteus maximus thickness indicates that the 45° leg press was not a sufficient hypertrophic stimulus, despite the pre-exhaustion of the quadriceps via leg extension. It is possible that the PreEx method impairs gluteus maximus maximum activation as suggested by Carpinelli (2010). Augustsson et al. (2003) found that PreEx with leg extension did not increase gluteus maximum activation during 45° leg press, and it seems that the pre-exhaustion of the knee extensors does not increase the activation of hip extensors.

It remains to be determined if the previous exhaustion of the gluteus maximum in the hip thrust exercise, an exercise with higher electromyographic activation of this muscle (Williams et al., 2018), could increase hip extensors hypertrophy. This strategy of promoting pre-exhaustion of synergic muscles, other than primary motors, proposed by Tan (1999), requires further investigations.

The PreEx group displayed increased maximal leg extension strength despite low exercise intensity (likely due to the specificity of the exercise). These data were supported in a study implementing one PreEx set of leg extension (20% 1RM) finding greater gains in leg extension strength (Aguiar et al., 2015). The authors showed that this previous set did not affect the performance of subsequent sets in the leg extension (75% 1RM), allowing an optimal combination of volume and intensity. To note, leg extension with 75% of 1RM was performed after 30 s of rest following the pre-exhaustive set.

Maximal strength in 45° leg press increased similarly between groups, suggesting that the metabolic stress generated by the isolation exercise may be sufficient to induce strength gains at similar magnitudes when associated with greater volumes of 45° leg press (American College of Sports Medicine, 2009). Some studies suggest that there is a necessity to increase training volume with low loads to obtain similar adaptations observed during high intensity RT (Mitchell et al., 2012; Figueiredo et al., 2017). However, the use of only one PreEx set with low load may be sufficient to increase the activation of additional muscle fibers (Schoenfeld, 2013). Although muscle hypertrophy can be obtained within distinct training zones in untrained individuals (Schoenfeld et al., 2016), the combination of low and high intensity in different exercises is not clearly investigated. Our findings indicate that despite the decrease in total training volume with the PreEx method, similar hypertrophic and strength gains can be achieved.

The present study has some limitations that should be considered. Participants had previous experience with RT, however, the investigators attempted to minimize the ceiling effect by only including those who had not trained in the 6 months previous to the study. Because of this, these results cannot be extrapolated for a highly trained population. The duration of the RT programs was relatively short and potentially limiting the hypertrophic potential, however, the results are encouraging that muscle growth is possible in the initial weeks of training initiation. Moreover, only 18 training sessions may be considered a limitation. The post-training values of muscle thickness from the present study are different from previous studies (Abe et al., 2000; Blazevich et al., 2007; Baroni et al., 2013), while anatomical points for measures and training variables were distinct. Furthermore, the intraclass coefficient for RF was low, and values should be interpreted with caution. Other measures of metabolic stress, training load monitoring, fatigue, and electromyographic activation were not included and would have given the authors more support for their theories. The number of subjects per group could also be higher, however, this did not appear to negatively impact the power of the analyses.

## Conclusion

We demonstrated that the PreEx training style can be included in RT programs. This style is efficient in producing hypertrophic and strength results, despite the low total volume. Furthermore, despite the similar increase in the lean mass in both groups, the TRT group observed reductions in fat mass compared with the PreEx method. The combination of low intensity single joint exercises with higher intensity multijoint exercises seems to be an efficient tool for selective hypertrophy of the quadriceps.

## Data Availability Statement

All datasets generated for this study are included in the article/supplementary material.

## Ethics Statement

This study was carried out in accordance with the recommendations of Ethics Committee of Federal University of Rio Grande do Norte, Brazil, with written informed consent from all subjects. All subjects gave written informed consent in accordance with the Declaration of Helsinki. The protocol was approved by the Ethics Committee of Federal University of Rio Grande do Norte (protocol number: 2.110.224).

## Author Contributions

TT, JP, and PD conceived and designed the research. TT, ES, and RT conducted the experiments. JP, LN, RM, and NS analyzed the data. RM, WS, and NS prepared the figures and tables. LN, TT, and BC drafted the manuscript. All authors interpreted the results of experiments, edited, revised, and approved the manuscript.

## Conflict of Interest

The authors declare that the research was conducted in the absence of any commercial or financial relationships that could be construed as a potential conflict of interest. The handling Editor declared a shared affiliation, though no other collaboration, with one of the authors, NS, at the time of the review.
